# FGF/FGFR-Dependent Molecular Mechanisms Underlying Anti-Cancer Drug Resistance

**DOI:** 10.3390/cancers13225796

**Published:** 2021-11-18

**Authors:** Jakub Szymczyk, Katarzyna Dominika Sluzalska, Izabela Materla, Lukasz Opalinski, Jacek Otlewski, Malgorzata Zakrzewska

**Affiliations:** Department of Protein Engineering, Faculty of Biotechnology, University of Wroclaw, Ul. F. Joliot-Curie 14a, 50-383 Wroclaw, Poland; jakub.szymczyk@uwr.edu.pl (J.S.); katarzyna.sluzalska@uwr.edu.pl (K.D.S.); zmaterla12@gmail.com (I.M.); lukasz.opalinski@uwr.edu.pl (L.O.); jacek.otlewski@uwr.edu.pl (J.O.)

**Keywords:** FGF, FGFR, drug resistance, cancer, cancer treatment, anti-cancer drugs

## Abstract

**Simple Summary:**

Deregulation of the FGF/FGFR axis is associated with many types of cancer and contributes to the development of chemoresistance, limiting the effectiveness of current treatment strategies. There are several mechanisms involved in this phenomenon, including cross-talks with other signaling pathways, avoidance of apoptosis, stimulation of angiogenesis, and initiation of EMT. Here, we provide an overview of current research and approaches focusing on targeting components of the FGFR/FGF signaling module to overcome drug resistance during anti-cancer therapy.

**Abstract:**

Increased expression of both FGF proteins and their receptors observed in many cancers is often associated with the development of chemoresistance, limiting the effectiveness of currently used anti-cancer therapies. Malfunctioning of the FGF/FGFR axis in cancer cells generates a number of molecular mechanisms that may affect the sensitivity of tumors to the applied drugs. Of key importance is the deregulation of cell signaling, which can lead to increased cell proliferation, survival, and motility, and ultimately to malignancy. Signaling pathways activated by FGFRs inhibit apoptosis, reducing the cytotoxic effect of some anti-cancer drugs. FGFRs-dependent signaling may also initiate angiogenesis and EMT, which facilitates metastasis and also correlates with drug resistance. Therefore, treatment strategies based on FGF/FGFR inhibition (using receptor inhibitors, ligand traps, monoclonal antibodies, or microRNAs) appear to be extremely promising. However, this approach may lead to further development of resistance through acquisition of specific mutations, metabolism switching, and molecular cross-talks. This review brings together information on the mechanisms underlying the involvement of the FGF/FGFR axis in the generation of drug resistance in cancer and highlights the need for further research to overcome this serious problem with novel therapeutic strategies.

## 1. Introduction

The development of resistance to pharmaceutical treatment is a common problem that affects a broad spectrum of diseases, in particular cancer. Despite current advances in medicine and the existence of many initially effective anti-cancer therapies, patients are often found to have cancer relapse, which is more malignant, invulnerable to treatment, and significantly correlates with poor prognosis [1]. Therefore, intensive research has been carried out for many years to develop new therapeutic strategies that may reduce the risk of recurrence of drug-resistant cancers [1,2]. A large range of mechanisms potentially involved in the emergence of chemoresistance exists, which severely hinders overcoming this problem. These mechanisms, often arising from DNA mutations and metabolism switching, include expression of efflux cell membrane transporters, drug inactivation, alteration in drug molecular targets, enhancing DNA repair machinery, epithelial-to-mesenchymal transition (EMT), or inhibition of apoptosis [1,3]. Another aspect is the diverse tumor microenvironment and heterogeneity of cancer cells, characterized by the formation of many subpopulations of cells with different drug sensitivity and the evolution of resistant clones [2]. It is supposed that this phenomenon may be of great importance in the recurrence of less sensitive or treatment-resistant cancers that may spread to other organs [1]. In recent years, particular attention has been paid to the involvement of growth factors and their receptors in processes leading to drug resistance, due to their biological functions and the frequent correlation of overproduction of these proteins with cancer progression [3,4]. EGF (epithelial growth factor), IGF (insulin-like growth factor), VEGF (vascular endothelial growth factor), and their receptors have been identified as key players in the response of cancer cells to cytotoxic drugs, but the exact mechanisms of this phenomenon have not been fully elucidated [5,6,7,8]. Recent studies suggest that fibroblast growth factors (FGFs) and their receptors (FGFRs) are also an important group of proteins in the development of drug resistance.

The FGF family includes 22 highly conserved proteins that interact with specific receptors (FGFR1-4) belonging to RTKs (receptor tyrosine kinases) [9]. The FGF binding forces the conformational changes of FGFRs, followed by receptor dimerization and transphosphorylation in the intracellular kinase domain [10]. This interaction is stabilized by heparan sulfate proteoglycans located on the cell surface, due to their high affinity to both FGFs and FGFRs [9]. Upon FGFR dimerization, its phosphorylated kinase domain recruits and activates adaptor proteins, including FRS2α (fibroblast growth factor receptor substrate 2), which in turn interacts with GRB2 (growth factor receptor-bound 2) [11]. Furthermore, activated GRB2 recruits SOS1 (son of sevenless 1) to activate the RAS/MAPK (rat sarcoma virus protein/mitogen-activated protein kinases) pathway, which includes ERK1/2 (extracellular signal-regulated kinase 1/2) and p38, or GAB1 (GRB2-associated binding protein 1) to activate the PI3K/AKT/mTOR pathway (phosphoinositide 3-kinase/AKT/mammalian target of rapamycin) [10,11]. Independently of the interaction with FRS2α, activated FGFR kinase domain triggers activation of other signaling pathways, such as JAK/STATs (Janus kinases/signal transducers and activators of transcription) and PLCγ/PKC (phospholipase Cγ/protein kinase C) pathways [11]. Downstream FGF/FGFR signaling regulates pivotal cellular processes such as proliferation, differentiation, migration, and apoptosis, which govern embryogenesis, organs development and the maintenance of homeostasis in adult tissues [9,11,12]. Given the role of FGFs and FGFRs in cell and tissue development and function, they have been rapidly linked to tumorigenesis and chemoresistance occurring during anti-cancer therapy [13,14]. Here, the relationship between the action of FGF/FGFR and the occurrence of drug resistance in cancer cells is presented and their specific mechanisms of action are proposed to be considered as targets for cancer treatment.

## 2. FGFs and Their Receptors in Cancer Progression

In the late 1970s and early 1980s, FGFs and their specific receptors began to be associated with tumors [15,16]. Baird and co-workers showed that antibodies directed to FGF significantly decreased tumor size from transplantable chondrosarcoma [17]. At the same time, *Fgf-3* gene was identified as a proto-oncogene in MMTV (mouse mammary tumor virus)-induced tumor in mice [18]. In the following years, the number of correlations of FGFs and FGFRs with tumors increased [10]. Currently, ample evidence points to a role of unusual occurrence of FGFs and/or their receptors in the progression of cancer, including breast, lung, prostate, colorectal, brain, and other cancers, which is usually associated with poor patient prognosis [10,13]. However, there are reports showing that FGF2/FGFR2 protein level in glioma and breast cancer tissue does not differ from that in non-malignant parental cells, or is even lower [19,20].

**Figure 1:**
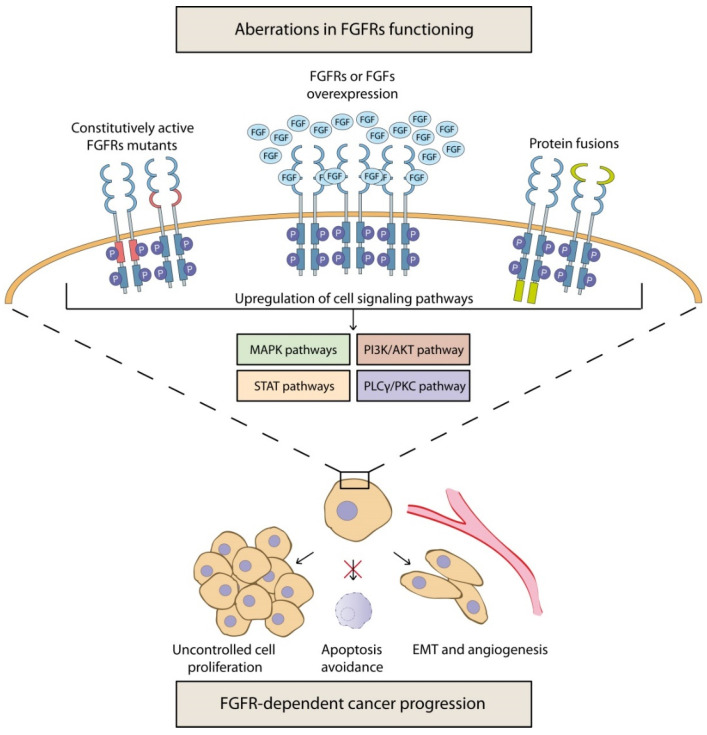
FGFRs-mediated mechanisms of cancer development and progression. Fibroblast growth factor receptors (FGFRs) and their natural ligands (FGFs) are involved in many biological processes, crucial for the proper operating of the cells and entire organism. However, many aberrations in FGFRs and/or FGFs genes may generate deregulations in the FGFRs/FGFs axis, which often upregulate downstream cell signaling and drive tumorigenesis. Activating mutations may lead to ligand-independent receptor dimerization and activation or creating the constitutively active kinase domains. The amplification of FGFRs or FGFs genes results in protein overexpression, which may also contribute to enhanced FGFRs-mediated cell signaling. Chromosomal translocations lead to the formation of fusion proteins that in some cases cause, similarly to activation mutations, receptor activation independently of FGFs presence. Regardless of the type of FGFRs dysfunction, the consequence is upregulated cell signaling that may drive cancer progression, through uncontrolled cell division, apoptosis avoidance, new blood vessel formation and/or EMT.

A number of dysfunctional aberrations, such as gene amplification, chromosomal translocations, or missense point mutations have been identified in FGFs and FGFRs genes in various cancers [12,21,22,23,24]. These anomalies often lead to overexpression of FGFs or their receptors, the formation of fusions of FGFRs with other proteins and/or the generation of a constitutively active kinase domain in FGFRs (Figure 1) [10]. This may result in imbalanced FGFRs-dependent cell signaling, which in turn facilitates uncontrolled cell proliferation, evasion of apoptosis, angiogenesis, and EMT (Figure 1) [10,11]. This may also cause genome instability, leading to further random mutations and the emergence of other mechanisms driving tumorigenesis [25]. However, it is still unclear whether the dysregulation of FGF/FGFR is directly responsible for carcinogenesis or whether the abnormalities, caused by genome instability, are site effects and only drive neoplastic progression. Nevertheless, FGFs and their receptors play an important role in cancer development and deregulated intracellular signaling may be largely responsible for the formation of malignant tumors, resistant to chemotherapy.

## 3. The Role of Cell Signaling Pathways in the Development of Anti-Cancer Drug Resistance

The increasing number of cases correlating FGF and FGFR expression in cancer cells with treatment failure and poor patient prognosis highlights the important role of these proteins in the cellular response to anti-cancer drugs. FGFs and FGFRs have been associated with resistance to several cytotoxic agents, such as paclitaxel, cisplatin, etoposide, 5-fluorouracil, doxorubicin, and others in various tumor types (Table 1) [26,27,28,29,30,31,32,33,34,35,36]. Most studies on the involvement of FGF proteins in the development of drug resistance involve FGF1 and FGF2, while there are a few reports on the role of other FGFs in this process, including FGF4, FGF5, FGF9, FGF10, FGF13, and FGF19 [37,38,39,40,41,42,43,44]. Overexpression of FGF receptors in cancer cells has also been observed with a concomitant reduction in response to protein kinase inhibitors (including RTKs) or endocrine therapy (Table 1) [39,45,46,47,48,49,50]. To investigate the involvement of FGF proteins and their receptors in the process of chemoresistance, particular attention has been paid to FGFR-dependent signaling pathways and their downstream targets, which can lead to deregulation of a number of biological processes, including apoptosis and metastasis.

Cancer cell signaling is a highly unpredictable “life-to-death” machinery due to the interference of multiple independent factors. Therefore drug-resistant tumors frequently exhibit deregulations in more than one signaling pathway, with growth factor-associated cascades often playing a significant role here [3,11]. A growing number of cases indicate that mutations in various signaling proteins, aberrant signal transduction, and abnormal cross-talks between different cascades are key problems in overcoming the after-treatment recurrence of more aggressive and resistant cancers.

### 3.1. MAPK Cascade

**Figure 2:**
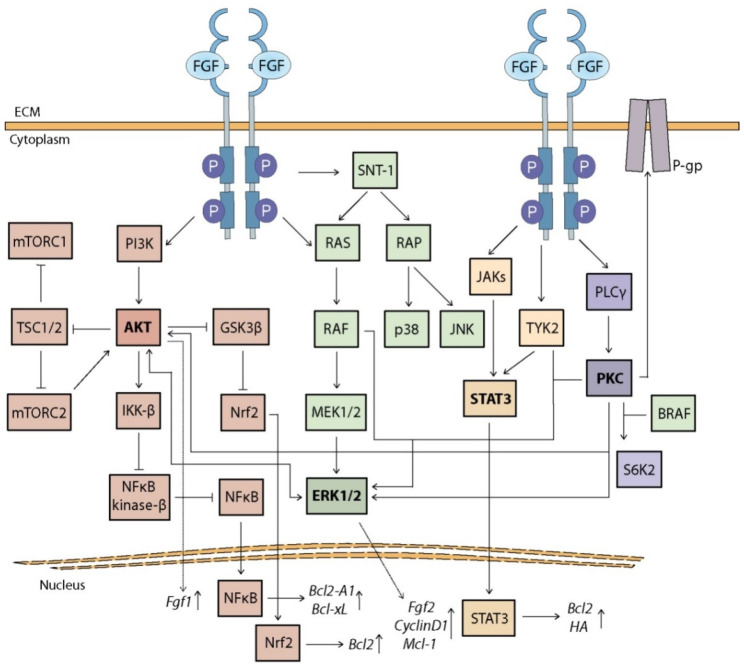
FGFR-dependent cell signaling in the development of drug resistance. Many drugs show reduced efficacy over time. Recent studies have linked this phenomenon to activation of FGFR and disruption of its downstream signaling including mutations of individual signaling proteins, impaired signal transduction and cross-talks between different cascades. The mode of action of PI3K/AKT in the development of chemoresistance is mainly based on the upregulation of pro-survival genes such as *Bcl2-A1* or *Bcl-xL* by activation of IKKβ or inactivation of GSK3β and further nuclear translocation of NFκB or Nrf2, respectively. Another mechanism is inhibition of TSC1/2 affecting protein synthesis. Activation of ERK kinases in MAPK pathway appears to be crucial in the acquisition of drug resistance, as it leads to increased expression of proteins associated with cell cycle progression (e.g., cyclin D1) and apoptosis control (e.g., Mcl-1). Another signaling pathway STAT, in particular STAT3, can directly affect the tumor microenvironment through increased hyaluronan (HA) synthesis, as well as the regulation of apoptosis through Mcl-1 or Bcl-xL. Finally, the PLCγ/PKC pathway is often complicit in other signaling cascades and is also able to directly activate multidrug resistant proteins such as P-gp.

Mitogen-activated protein kinases (MAPKs) are an integral part of signaling pathways with high mitogenic and pro-survival potential and include three main families: ERK family as mitogen-responsive and JNK and p38 kinase families as stress-responsive [59]. The MAPK pathways, initiated by G proteins (RAS for ERK and RAC, RHO or RAP for JNK and p38), are three-tiered kinase cascades that act through phosphorylation of subsequent kinases (MAPKKK, MAPKK, and MAPK) (Figure 2) and regulate many biological processes, such as embryogenesis, cell differentiation, proliferation, and cell death [60]. All three MAPK families have been reported to control apoptosis in response to anti-cancer drugs [59]. However, the activity of the RAS/RAF/MEK/ERK pathway is most commonly correlated with FGF/FGFR-dependent drug resistance in many types of cancer [37,47,53,61]. ERK upregulation caused by increasing FGFR3 level in HNSCC (head and neck squamous cell carcinoma) cells led to an increase in FGF2 expression, which correlated with reduced sensitivity to bevacizumab (Figure 2) [53]. In colorectal cancer (CRC) cells, resistance to irinotecan, a topoisomerase I inhibitor, was dependent on FGF2 and FGF9 expression followed by MAPK pathway activation [37]. FGF9 seems to have a key role in irinotecan resistance as it correlates with other identified genes, such as *Prkacb* (cAMP-dependent protein kinase catalytic subunit beta) and *Macom* (MDS1 and EVI1 complex locus protein EVI1), and also with *Ffg2* and *Pla2g4c* (cytosolic phospholipase A2γ) [37]. Hepatic stellate cells (HSC), but not HCC cells, produce FGF9 which correlates with poor patient survival [42]. However, the exogenous addition of FGF9 in HCC cells activated ERK and JNK, and led to a decrease in sorafenib sensitivity, which suggests the FGF-dependent HSC-HCC cross-talk in liver cancers [42]. The reactivation of ERK seems to be crucial for the resistance to BRAF inhibitors in melanoma cells with BRAF-V600E mutations, and one of the mediators is FGFR3 [47]. In BRAF/MEK inhibition-resistant cancer cells carrying BRAFV600E mutation, the dual MAPK inhibition drives the overexpression of FGF1 followed by the FGFR activation and the reactivation of ERK [62]. The addition of FGFR inhibitors re-sensitized cells to combination treatment with vemurafenib and cobimetinib, BRAF and MEK inhibitors, respectively [62]. Resistance to trametinib (MEKs inhibitor) treatment via FGFR1-dependent activation of ERKs and AKT was also observed in lung and pancreatic cancer cells with mutated KRAS [61]. Similarly, reactivation of ERKs by exogenous FGF4 in HER2-positive breast cancer led to the resistance to dual HER2 inhibition [43].

Another concern is the development of resistance to tamoxifen, an antagonist of estrogen receptor (ER), commonly used in breast cancer therapy [1]. In vivo studies in ER-positive breast cancer (MCF-7 cells) showed that resistance to tamoxifen treatment is dependent on reactivation of ERK1/2 and p38 kinase [8]. Zhang and co-workers showed that overexpression of FGF1 promotes tumor growth in breast cancer treated with tamoxifen [48]. A similar effect was observed for FGF2 and FGF4 [63,64]. Administration of exogenous FGF1, but not EGF, reduced the inhibitory effect on ERK1/2 activation by MEK inhibitors and reduced the inhibition of tumor growth by the ER antagonist ICI 182780 in MCF-7 cells [65]. Other studies showed that amplification of FGFR1, FGFR2, or FGFR3 in ER-positive human breast cancers correlates with concomitant resistance to estrogen-related therapy [65,66,67,68,69]. The mechanism is probably based on prolonged ERK1/2 activation after FGF/FGFR stimulation [65,66]. It has been suggested that it depends on RAP1 (RAS-proximate-1 or Ras-related protein 1) and SNT-1 (Suc1-associated neurotrophic factor-induced tyrosine-phosphorylated target)/FRS2 rather on RAS or RAF-1 proteins (Figure 2) [65]. Stimulation with FGFs causes SNT-1/FRS2 phosphorylation and its binding to FGFRs via the phosphotyrosine-binding domain, which consequently leads to activation of downstream signaling pathways through its interaction with adaptor proteins GRB2, GAB1 and SOS1 [65]. Inhibition of the interaction between SNT-1/FRS2 and FGFRs decreases MAPK, PI3K, and mTOR activity, leading to a reduction in anti-estrogen resistance induced by FGF1 stimulation [69]. Furthermore, FGFR1 signaling activation decreased progesterone receptor expression [69]. FGF2/FGFR-dependent ERK activation induced cyclin D1 expression, which activates key mediators of cell cycle progression, cyclin dependent kinases 4 and 6 (CDK4, CDK6), in ER-positive breast cancer (Figure 2) [64]. Turner and co-workers have shown that stimulation with FGF2 led to the development of resistance to tamoxifen in breast cancer cells with elevated expression of FGFR1 [66]. It has been suggested that FGF2/FGFR1 signaling is essential for overcoming tamoxifen action, and this process has been associated with high activity of MAPK and AKT cascades as well as increased level of cyclin D1 [66].

### 3.2. PI3K/AKT Cascade

Protein kinase B (PKB), also known as AKT, is a major downstream effector of PI3K and one of the main perpetrators associated with the resistance to various anti-cancer agents, including cisplatin, paclitaxel, etoposide, RTKs inhibitors, as well as radiation [70,71,72,73,74]. Activated by PI3K and mTORC2 (mammalian target of rapamycin complex 2), AKT directly or indirectly exerts control over the activity of various intracellular processes, including apoptosis, through inactivation of proapoptotic BAD (Bcl-2 antagonist of cell death), BIM (Bcl-2-like protein 11), pro-caspase 9, and/or FOXO (forkhead box protein O1) proteins or phosphorylation of MDM2 (mouse double minute 2 homolog) followed by increased p53 (cellular tumor antigen p53) degradation; protein synthesis, by inhibition of TSC1/2 (tuberous sclerosis complex 1/2, mTOR inhibitors) and subsequent activation of mTORC1 (mammalian target of rapamycin compex 1); and cell cycle regulation through inhibition of the cyclin-dependent kinase inhibitors, p21 and p27, or glycogen synthase kinase 3β (GSK3β), which prevents cyclin D1 degradation [75]. AKT can also regulate angiogenesis and cell migration, which in turn can lead to EMT in cancer cells [76]. The PI3K/AKT pathway has been indicated as a major player in FGF/FGFR-dependent tumor progression with a significant role in the regulation of apoptosis and the development of chemoresistance [57,67,77,78]. The interaction of FGF2 and FGFR1, through activation of the PI3K/AKT pathway, mediates cell survival, proliferation, motility, and consequently resistance to cytarabine and paclitaxel treatment [55,79].

The use of anti-FGFR1 antibody reduces AKT phosphorylation, inhibits tumor growth and restores drug sensitivity both in vitro and in vivo [55]. FGF/FGFR activity plays also an important role in acquired resistance to EGFR inhibitors [80,81,82,83]. Overexpression of FGFR1 in resistant to gefitinib (EGFR inhibitor) NSCLC (non-small-cell lung cancer) cells led to increased activation of AKT and mTOR, whereas FGFR1 inhibition decreased phosphorylation of both kinases and re-sensitized cells to gefitinib [54,83]. The investigation into the role of the PI3K/AKT pathway in FGF2-dependent resistance to etoposide, 5-fluorouracil, camptothecin, and the C2 ceramide analogue in breast cancer cell lines (MCF-7, T47-D, and BT-20) revealed subsequent AKT-dependent stimulation and translocation of nuclear factor-κB (NFκB) to the nucleus via activation of IKK-β (inhibitors of NFκB kinase-β), but not on the MAPK pathway [30]. Further studies have revealed that FGF2/PI3K-dependent translocation of NFκB mediates transcriptional upregulation of pro-survival *Bcl2-A1* (Bcl-2-related protein A1) and *Bcl-xL* (Bcl-2-like protein 1) genes (Figure 2) [84]. Gao and co-workers demonstrated that FGF19/FGFR4 signaling in hepatocellular carcinoma (HCC) cells is one of the main resistance mechanisms to sorafenib, a multikinase inhibitor inducing the ROS (reactive oxygen species)-associated apoptosis [39]. Further research revealed that overexpression of FGF19 induced by endoplasmic reticulum stress in HCC cells leads to increased resistance to apoptosis through the inactivation of GSK3β and subsequent nuclear translocation of Nrf2 (nuclear factor E2-related factor 2) (Figure 2) [85]. FGFR-mediated activation of the PI3K/AKT pathway is also observed in cancer cells during BRAF (serine/threonine-protein kinase B-raf) or MEK (mitogen-activated protein kinase kinase) inhibitors treatment [62,86,87]. In neural stem cells, transactivation of FGFR1 by Notch2 (neurogenic locus notch homolog protein 2) led to GSK3 inhibition via AKT, but independently from mTOR activation, resulting in a reduced response to etoposide [58]. Knock-down of FGF2, but not EGF or insulin, re-sensitized these cells to treatment. Interestingly, inhibition of the mTOR pathway, but not the PI3K pathway, in triple-negative breast cancer cells led to increased FGF1 and Notch1 expression, enhanced FGFR1 activation and the formation of a resistant cancer stem cell-like population [88].

Recently, autocrine FGFR activation has been correlated with HES1 (hairy and enhancer of split-1) expression and AKT-dependent cell proliferation in endometrial cancers insensitive to Notch inhibition [89]. HES1 has been also identified as a potential driver of anti-cancer drug resistance, in addition to upregulation of cancer cell proliferation and migration [90]. The development of resistance to the BRAF inhibitor (vemurafenib) in melanoma cancer cells was correlated with the induction of transcription and secretion of FGF1 through enhanced activity of PI3K/AKT pathway and FRA1 (Fos-related antigen 1) (Figure 2) [87]. Expression of FGF2 by endothelial cells promoted prostate cancer cells proliferation and led to the acquisition of docetaxel resistance via activation of the AKT/mTOR pathway and upregulation of the erythroblast transformation specific related gene [91]. In ovarian cancer, the FGFR/PI3K/AKT pathway has been implicated in the development of cisplatin resistance induced by the GLT8D (glycosylotransferase 8 domain containing2) protein through the interaction with FGFR1 and the subsequent signaling activation [92].

Additionally, the FGF7/FGFR2 axis has been identified as driving tamoxifen resistance in breast cancer cells (T47-D, MCF-7). It has been shown that FGFR2 activation counteracted the effect of tamoxifen on ER stabilization and that the acquisition of resistance to the aforementioned drug was promoted by the PI3K/AKT cascade and not by MAPKs, further targeting ER-Ser167 and Bcl-2 expression [51]. They showed that FGFR2 signaling promoted ER ubiquitination and suggested that the ER degradation/turnover is the main mechanism responsible for the suppressed cellular response to tamoxifen [51]. This observation is in agreement with previous work confirming that CUEDC2 (a ubiquitin-binding motif-containing protein which regulates ER degradation) is the molecule that leads to resistance to tamoxifen-based therapy [93].

### 3.3. STAT Cascade

Another important group of proteins activated by extracellular signals transmitted by RTKs and involved in cancer progression and survival are signal transducers and activators of transcription (STATs), in particular STAT3 and STAT5 [94]. Upon activation by receptor-bound JAK1-3 (Janus kinases) and TYK2 (tyrosine kinase 2), phosphorylated STATs form homo- or heterodimers and translocate to the nucleus, where they act as transcription factors [94,95]. STATs directly target genes that regulate cell cycle, such as *cyclin D1*, *p21*, *c-myc*, and apoptosis, such as *Bcl-xL* and *Mcl-1* (myeloid cell leukemia sequence 1), and thus may also affect cellular response to anti-cancer drugs [95]. FGF/FGFR-dependent STAT3 activity has been correlated with resistance to doxorubicin, 5-fluorouracil, cisplatin, paclitaxel, and MEK/BRAF inhibitors in several cancers [52,96]. Activation of FGFR1 induces hyaluronan (HA) synthesis via STAT3 pathway and causes accumulation of HA in extracellular matrix (ECM) of breast cancer cells (Figure 2) [96]. Inhibition of HA synthesis and/or accumulation by STAT3 inhibition reduces cell migration and proliferation, and partially reverses resistance to doxorubicin [96]. Overexpression of FGFR3 decreases apoptosis in multiple myeloma cells, with concomitant increase in STAT3 phosphorylation and Bcl-xL expression (Figure 2) [97]. Another study demonstrated that resistance to 5-fluorouracil and oxaliplatin in CRC cells was associated with the upregulation of FGFR4 and subsequent STAT3 activity [52]. Additionally in CRC, the FGFR2/JAK/STAT3 pathway promotes the expression of programmed cell death ligand 1 (PD-L1), a transmembrane protein associated with reduced T-cell proliferation [98].

Recently, studies in EGFR-positive cancer cells have shown that sustained activation of STAT3, due to enhancement of its binding to FGFR1, plays a key role in the acquisition of resistance to EGFR inhibitors [99]. Moreover, STAT3 activation was independent of gp130/JAK activity or HER2/EGFR heterodimer formation and administration of PD173074 led to suppression of STAT3 activation and inhibition of cancer cell proliferation [99]. Interestingly, in the osteosarcoma U2OS cells, FGF2-dependent drug resistance was determined by activation of JAK1, JAK2, and TYK2, but not STATs activation [100].

### 3.4. PLCγ/PKC Cascade

Finally, phospholipase Cγ (PLCγ) is also a very important signaling mediator involved in many biological processes, acting by cleavage of PIP2 (phosphatidylinosi-tol-4,5-bisphosphate) into IP3 (inositol-1,4,5-triphosphate) and DAG (diacyloglycerol), and regulating Ca^2+^ channels and PKC activity [101]. Aberrations in the PLCγ/PKC pathway also contribute to the development and progression of many types of cancers, but their role in the drug sensitivity is not fully elucidated [101]. PLCγ acts primarily by PKC activation as a regulator and/or alternative activator of other pro-survival proteins, such as AKT or RAS (Figure 2) [5,67,101]. Activated PKC can also phosphorylate multidrug resistance proteins, such as P-gp (P-glycoprotein 1, also known as MDR1 or ABCB1) (Figure 2) [31]. It has been observed that increased expression of FGF2, but not FGF1, in tumors with reduced sensitivity to paclitaxel also correlates with increased level of P-gp [31]. In FGF/FGFR-dependent mechanisms of drug resistance involving other signaling pathways, co-involvement of the PLCγ/PKC cascade has been observed, indicating an important role for PLCγ in the development of molecular cross-talks necessary for cell survival [53,67,102,103].

## 4. Signals from the Tumor Microenvironment

It is well established that the complex tumor microenvironment can play an important role in cancer progression, metastasis, as well as in gaining the resistance to treatment. Its components including ECM, immune cells, blood vessels, cytokines and growth factors, and non-epithelial cells, especially cancer-associated fibroblast (CAFs), provide additional pro-survival stimuli to adapt to treatment and evade therapies. Activated fibroblasts acquire an invasive phenotype and promote tumor growth and proliferation via paracrine and autocrine pathways, further fueled by the immune response [104,105].

Members of FGF family (including FGF1, FGF2, FGF4, FGF5, FGF6, FGF7, and FGF9) were reported to be secreted by CAFs [51,106,107,108] and FGFR2 was shown to be a key mediator of tumor niche-derived signals that are responsible for the acquisition of tamoxifen resistance [51]. In addition, FGF2 secreted by CAFs was found to contribute to lung cancer cells growth through overexpression of *Tgfb*, *Mmp7*, *Fgf2*, *Fgf9*, enhanced collagen synthesis, and increased expression of inflammatory cytokines such as *Csf1*, *Cxcl12,* and *Ccl2* [109]. Furthermore, FGF1 promotes tumor-niche fibroblasts to express and secrete HGF (hepatocyte growth factor), a mediator of angiogenesis and cell motility, and an important tumor-resistant factor in melanomas [87].

Another study showed that HGF secreted by CAFs induced FGF2 secretion by HNSCC in order to drive glycolysis for extensive use of glucose for their growth and survival [110]. Additionally in pancreatic adenocarcinoma, CAFs secrete FGF2, which enhances cancer survival by increasing CXCL8 level [111]. In HER-positive breast cancer, the secretion of FGF5 by CAFs leads to FGFR2 activation in cancer cells and acquisition of resistance to trastuzumab and lapatinib (EGFR/HER2 inhibitors) through FGFR2/c-Src-mediated HER2 transactivation [112]. Furthermore, FGF2 secreted by CAFs stimulated cell migration and invasiveness in a breast cancer cell line (MDA-MB-231), which could be inhibited by an FGF2-neutralizing antibody [113].

## 5. Cross-Talks between FGF/FGFR Signaling Pathways in Cancer

Due to the frequent involvement of more than one pathway and the formation of molecular cross-talks between them, it is difficult to pinpoint the main mechanism responsible for the impaired cellular response to anti-cancer treatment. For instance, RAS/MAPK and PI3K/AKT pathways may alternately activate when one pathway is inhibited, thereby preventing apoptosis in cancer cells [114]. Clinical studies indicate that parallel inhibition of the PI3K/AKT and RAS/MAPK pathways can significantly improve treatment efficacy, especially in advanced cancers with genetic alterations in these pathways [115]. Interestingly, also in doxorubicin-treated non-cancerous NIH3T3 cells, the PI3K/AKT and p38 MAPK pathways were transiently activated and their chemical inhibition accelerated and enhanced drug-induced apoptosis, whereas the ERK and JNK pathways were continuously active and their inhibition repressed the apoptotic function of doxorubicin [116]. Moreover, AKT activity has been shown to negatively affect long-term ERK phosphorylation followed by PARP (poly (ADP-ribose) polymerase) cleavage and caspase activation during doxorubicin-induced cell death [116]. In KRAS mutant cancer cells, only inhibition of both the PI3K/AKT and MEK pathways resulted in complete inactivation of mTOR and increased cell death (Figure 2) [117]. Increased phosphorylation of both AKT and ERKs was commonly observed in cancer cells treated with RAS/MAPK pathway inhibitors, suggesting the importance of the PI3K/AKT pathway in reactivating ERKs and reducing the cytostatic effect of the inhibitors (Figure 2) [61,62,118]. In lung and pancreatic cancer cells with mutated KRAS, treatment with trametinib, a MEK inhibitor which acts downstream of KRAS to suppress MAPK cascade, led to a compensatory response through activation of FGFR1 and subsequent phosphorylation of FRS2, thus resulting in alternative signal transduction and generating adaptive drug resistance [61]. Inhibition of FGFR1 in combination with trametinib treatment induced cell death in KRAS-mutant cancer cells [61]. Another study showed that in HNSCC cells, co-inhibition of MEK and FGFR3 activity reduced AKT and ERKs phosphorylation, which in turn led to increased DNA fragmentation, caspase 3 cleavage, and reduced tumor growth in vivo [118].

One of the multi-functional mediators of linking different signaling pathways is the aforementioned PLCγ/PKC pathway. PLCγ downstream signaling has been shown to activate MAPK and PI3K pathways in the development of resistance to estrogen-related therapy (Figure 2) [67]. Additionally, increased FGF2 expression in bevacizumab-resistant HNSCC cells, accompanied with upregulation of PLCγ, ERKs, and AKT, correlated with the reduced sensitivity to the bevacizumab treatment [53]. Other studies indicate an important role for the interaction of MAPKs and S6K2 (ribosomal p70 S6 kinase 2) in FGFs-dependent cancer cells proliferation and survival [102,119]. As a consequence of FGF2 stimulation, PKCε complex formation with BRAF and S6K2, but not with S6K1 and RAF-1, induced drug resistance in SCLC (small cell lung cancer) cells, HEK293 and U2OS cells (Figure 2) [100,102]. FGF2 was also found to mediate the interaction of TYK2 with PKCε and BRAF, leading to full phosphorylation of ERK1/2, MCL-1 activation and inhibition of apoptosis (Figure 2) [100]. Furthermore, another phospholipase, PLA2G4C (cytosolic phospholipase A2γ), has been linked together with FGF9 and the MAPK pathway to irinotecan resistance in CRC cells [37].

## 6. Dysregulation of Apoptosis in Cancer by FGFs/FGFRs System

One of the most essential processes for the functioning of cells and the whole organism is apoptosis, a programmed cell death, controlled by two main pathways: extrinsic (death-receptor pathway) and intrinsic (mitochondrial-mediated pathway) [120]. Increased survival of cancer cells by avoiding apoptosis is one of the most potent mechanisms of tumor progression and drug resistance [2]. A majority of anti-cancer drugs act by activating apoptosis mediated by cell cycle inhibition, DNA damage, immune surveillance, and other cellular stresses [2]. Therefore, alteration of pathways involved in cell death and imbalance between activators (such as BAD or BAX) and inhibitors (such as Bcl-2 or Bcl-xL) of apoptosis often leads to reduced drug sensitivity and the development of chemoresistance in tumors [2,120].

**Figure 3:**
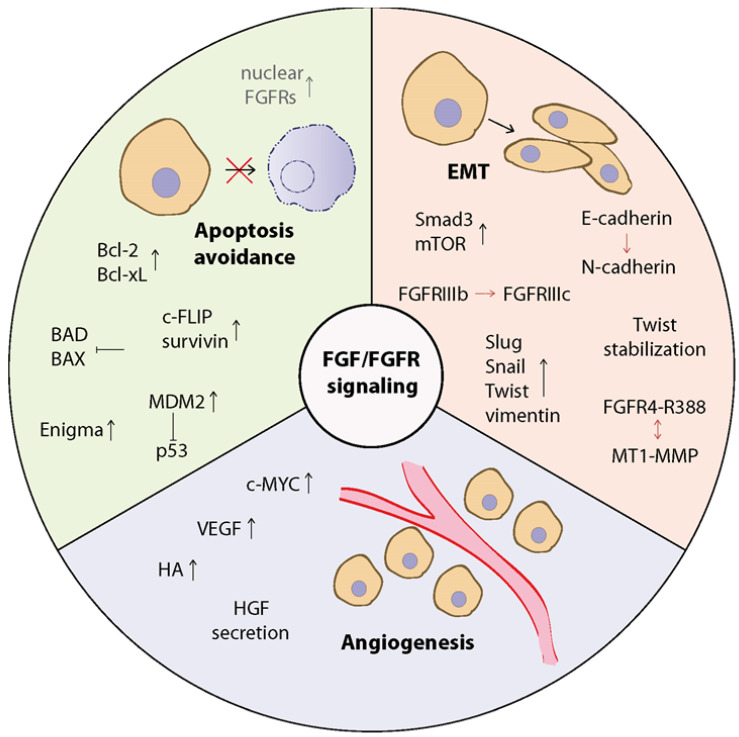
Involvement of FGF/FGFRs in cellular processes during the development of drug resistance. The active FGF/FGFR complex leads to avoidance of apoptosis through increased expression of apoptosis inhibitors (Bcl-2, Bcl-xL), inhibition of its activators (BAD, BAX) or activation and stabilization of MDM2, with consequent increased ubiquitination and degradation of p53. Additionally, p53 degradation can be enhanced by the formation of a ternary complex of p53, MDM2, and Enigma protein. Another mechanism of action is indirect inhibition of caspase 3/7 and caspase 8 by increasing the expression of survivin or c–FLIP, respectively. It is also likely that increased nuclear localization of FGFRs may be associated with enhanced survival of cancer cells and development of drug resistance. FGF/FGFR (especially involving FGF2) also promotes angiogenesis through increased expression of VEGF, HA, and c-MYC, and enhanced secretion of HGF. EMT, in turn, is a consequence of activation of the MAPK signaling pathway leading to stabilization of Twist, switching of FGFR isoforms from IIIb to IIIc, switching of cadherins (from E-cadherin to N-cadherin), upregulation of mesenchymal markers (such as Twist and vimentin) and transcription factors (Slug and Snail), and activation of downstream signaling proteins (such as Smad3 and mTOR). Furthermore, the FGFR4-R388 mutant can interact with matrix metalloproteinases (e.g., MT1-MMP), proteins involved in tumor invasion.

The action of FGFs and their receptors is also involved in the regulation of apoptosis, mainly through the activation of downstream signaling, such as PI3K/AKT, MAPKs, or STATs [10,97,121]. Several studies have shown that FGF2 regulates apoptosis through upregulation of Bcl-2, Bcl-xL, Mcl-1, and XIAP (X-linked inhibitor of apoptosis protein) [78,84,100,102,121,122]. In addition, Sun and co-workers have reported that FGF2 increases the expression of survivin, an anti-apoptotic protein that acts by inhibiting caspase 3 and 7 and linked this to the PI3K/AKT pathway in HCCs [78]. FGF2 has also been found to increase mitochondrial localization of Bcl2-A1 and Bcl-xL proteins via the PI3K/NFκB pathway [84]. Other work has indicated that increased levels of Bcl-xL and XIAP correlate with FGF2-dependent upregulation of PKCε [102]. FGF2 was also shown to protect SCLC cells from etoposide-induced apoptosis through upregulation of Bcl-xL and Bcl-2 at the translational level via the MEK pathway (Figure 3) [121]. On the other hand, the action of FGF1 or FGF2 was associated with inhibition of pro-apoptotic proteins, indicating a dual mode of action of FGFs in inhibiting apoptosis [121,123,124]. FGF2 has been shown to prevent cisplatin-induced activation of p53 by increasing MDM2 expression and block etoposide-triggered induction of BAD (Figure 3) [123]. FGF2 can also lead to MDM2 stabilization and enhanced p53 degradation by increasing the transcription of Enigma protein, which directly interacts with MDM2, forms a ternary complex with p53, and prevents MDM2 self-ubiquitination (Figure 3) [125]. It has been noted that only intracellular and not extracellular FGF1 affects p53-dependent apoptosis by increasing MDM2 expression [124]. However, it is known that exogenous FGF1 and FGF2 can translocate into the cytoplasm and cell nucleus independently of FGFRs activation, which is correlated with increased cell survival under stress conditions [126,127].

Inside the cell, FGF1 and FGF2 interact with many intracellular proteins, including p53, HSP90 (heat shock protein 90), CK2 (casein kinase 2), and others involved in the regulation of cell signaling, cell cycle, and apoptosis, which may contribute to the acquisition of drug resistance [128]. In PC12 cells, nuclear localization of FGF1 determines its anti-apoptotic effect, whereas in ovarian cancer cells FGF1 affects mitochondrial localization of p53 and reduces etoposide- and cisplatin-induced apoptosis (Figure 3) [56,129]. Of note, the nuclear FGF2 was associated with doxorubicin resistance in triple negative breast cancers with high level of DNA-dependent protein kinase, which is responsible for repairing double-stranded breaks in DNA [130]. Knockdown of FGF2 restored sensitivity to doxorubicin treatment. Furthermore, also nuclear localization of the FGF receptor has been associated with cancer progression and invasiveness [131,132,133]. In NSCLC cells, the importance of EGFR, another RTK, localized in the nucleus has been confirmed in the development of resistance to cetuximab, suggesting that FGFRs nucleocytoplasmic transport may also contribute to the development of drug resistance [134]. Treatment of patients with ER+/FGFR1-amplified breast cancers with letrozole, an aromatase inhibitor, increased the expression of FGFR1 and FGFs, as well as the nuclear localization of FGFR1 and ERα [135]. Estrogen deprivation led to an interaction between FGFR1 and ERα in the nucleus of cancer cells and subsequent regulation of ER-dependent genes transcription. This FGFR1:ERα interplay was abolished by the administration in the presence of TKIs (tyrosine kinase inhibitors) inhibitors or a kinase-dead FGFR1 mutant [135]. Another study has shown that doxorubicin treatment led to the formation of resistant cancer cell clones characterized by upregulated FGFR4 and *Bcl-xL* genes expression [136]. FGFR4 knockdown in these cells reduced ERK1/2 activity and Bcl-xL expression and resulted in re-sensitization of the cells to the drug [136]. Moreover, siRNA-mediated silencing of FGFR4 in CRC cells decreased the expression of Bcl-2 and c-FLIP (FLICE-like inhibitory protein), an inhibitor of caspase 8, while reducing STAT3 activity, which in turn induced caspase-dependent apoptosis (Figure 3) [52].

## 7. Role of FGFs/FGFRs Axis during Cancer-Associated Angiogenesis

Angiogenesis, the formation of new blood vessels from existing vasculature, is an essential process in mammalian tissues, e.g., during embryogenesis or wound healing [137]. However, this process is also involved in the development of many pathological conditions including carcinogenesis, as it supplies tumors with nutrients, oxygen, and cytokines, and promotes neovascularization, growth, invasion, and metastasis of tumors [138,139,140]. Several growth factors are involved in the regulation of angiogenesis, both physiological and pathological, e.g., VEGF, PD-ECGF (platelet-derived endothelial cell growth factor), FGF2, which act as major angiogenic agents by stimulating endothelial cells growth and motility [137,141].

The FGF/FGFR signaling pathway is often disrupted during malignant tumor progression and there are several examples of its impact on tumor-associated angiogenesis [138,140,142,143,144,145]. Huang and co-workers showed that in addition to the proliferative effect on hepatoma cells, FGFR1 ectopic expression also upregulates VEGF expression, which in turn enhances angiogenesis necessary for later stages of cancer progression (Figure 3) [142]. Previous studies have shown that both VEGF and FGF2 act synergistically in tumor-driving angiogenesis [146,147]. On the other hand, in VEGFR-positive tumors, expression of FGF2 and FGF receptors was correlated with resistance to anti-VEGF therapy [148]. Another group reported that co-treatment of a T lymphoma cell line with HA and doxorubicin stimulated angiogenesis and this effect was FGF2-dependent rather than VEGF-dependent [149]. This revealed that the mechanism of negative response to doxorubicin is not only due to drug efflux, but also to angiogenesis [149]. Schönau and co-workers evaluated the effect of 5-fluorouracil-resistant colon carcinoma cells on endothelial cells growth [144]. The stimulatory effect of FGF2 released from cancer cells was higher in macrovascular endothelial cells than in microvascular cells [144]. Interestingly, the level of chemoresistance of cancer cells did not differentially affect growth of endothelial cells in vitro [144]. An increase in FGF2 and FGFR3 expression was also found in HNSCC cells resistant to bevacizumab, a monoclonal antibody targeting VEGF used as an anti-angiogenic drug [53]. Simultaneous inhibition of VEGFR2 and FGFs blocked angiogenesis and tumor growth in pancreatic cancer resistant to anti-VEGFR treatment [150]. Furthermore, elevated levels of FGFR1 and FGFR3 associated with tumor progression and drug resistance, also correlated with expression of c-MYC, another protein responsible for tumor angiogenesis (Figure 3) [151,152,153]. Inhibition of FGFR3 in a urothelial cancer cell line or FGFR1 in a lung cancer cell line resulted in decreased c-MYC protein level [153,154].

## 8. Contribution of FGFs/FGFRs to EMT

EMT results in the transformation of epithelial cells into cells with a mesenchymal phenotype [155]. In this process, epithelial cells lose cell polarity, cell–cell adhesion, and acquire the ability to migrate. EMT is involved in embryonic development, but also plays an important role in cancer pathogenesis, as cells become able to move to distant regions, leading to tumor invasion and metastasis [155,156]. Cells that have undergone EMT have lower expression levels of epithelial markers, such as E-cadherin, a cell adhesion molecule (CAM), while they exhibit higher expression of mesenchymal markers, including vimentin and fibronectin, as a result of upregulation of transcription factors from the zinc finger family, e.g., Twist, Slug, Snail, E12/E47 (E2A-encoded transcription factors), EF1/ZEB1 (elongation factor 1/zinc finger E-box-binding homeobox 1), and SIP1/ZEB2 (Smad interacting protein 1/zinc finger E-box-binding homeobox 2) [157,158]. Furthermore, during EMT, a decrease in E-cadherin level is accompanied by increased level of N-cadherin, another CAM commonly expressed by mesenchymal cells, and this process is known as cadherin switching [158,159]. Most EMT processes are regulated by extracellular matrix components, cytokines, and growth factors, among which FGFs affect various functions by binding to FGF receptors [160,161,162]. One of the main inducers of EMT in many types of cancer is FGF2, which activates EMT through the MEK/ERK signaling pathway (Figure 3) [157]. In lung cancer cell lines (H1581 and DMS114), FGF2-dependent activation of the FGFR1/ERKs pathway led to upregulation of SOX2 (Sry-related HMG box 2), which in turn promoted EMT and cell migration [162]. Moreover, it has been suggested that high expression of both FGFR1 and SOX2 is associated with shorter survival of lung cancer patients [162]. However, SOX2 may be also regulated by the PI3K/AKT pathway, which is related with drug resistance in lymphoma [163]. Importantly, activation of epithelial FGFR1/2 by paracrine FGFs promotes tumor progression and induces EMT in vivo, which is associated with a switch of FGFR isoforms from IIIb to IIIc (Figure 3) [164,165,166,167,168]. In metastatic cancer cells, increased level of FGFR1c was triggered by transcription factor FOXC1, one of the predictors of EMT (Figure 3) [169]. Transformation of FGFR2b to FGFR2c also inhibited E-cadherin expression but increased vimentin expression (Figure 3) [168]. Furthermore, N-cadherin-induced tumor invasiveness was increased by FGF2, through stabilization of the FGF2-FGFR1 complex and sustained activation of the MAPK pathway [158]. Interestingly, the expression of FGFR2b in PC-3 cells caused the decrease in level of EMT markers [170]. EMT has been also found to contribute to the chemoresistance of cancer cells [171,172].

There are several examples demonstrating that upregulation of EMT markers can affect the drug sensitivity of cancer cells, e.g., in prostate cancer patients to docetaxel treatment or in NSCLC patients to cisplatin-based chemotherapy [81,157,170]. In lung and breast cancer cell lines resistant to ErbB inhibitors, the increased expression of FGFR1 and/or FGF2 accompanied EMT [81,82,83,173,174,175]. Another study showed that TGFβ (transforming growth factor β) and FGF2 efficiently induced EMT in human lung adenocarcinoma cell lines: in PC-9 cells via the Smad3 pathway, and in HCC-827 cells through Smad3, MEK/ERK, and mTOR pathways resulting in reduced sensitivity to gefitinib in both cell lines and to cisplatin only in HCC-827 cells (Figure 3) [81]. Inhibition of FGF2 or FGFR1 in a pemetrexed-resistant lung adenocarcinoma cell line resulted in downregulation of vimentin and Slug level, reversed EMT cell morphology, and partially restored sensitivity to pemetrexed [176]. Prifenidone and nintedanib reversed EMT-related chemoresistance induced by TGFβ and FGF2 in human lung adenocarcinoma cells [157]. Breast cancer cells resistant to lapatinib with a post-treatment mesenchymal phenotype showed increased level of FGFR1IIIc and FGF2 [177]. Interestingly, FGFR1 expression was induced by the EMT transcription factor Twist, while the following FGFR1-induced MAPK pathway stabilized Twist, and thus maintained the drug resistance (Figure 3) [177]. In afatinib-resistant lung cancer cell line, among EMT-related markers, only knockdown of Twist resulted in a complete downregulation of FGFR1, with concomitant inhibition of AKT and ERKs phosphorylation, which re-sensitized cells to afatinib [174]. Furthermore, FGFR1 was shown to contribute to acquired resistance to mesenchymal–epithelial transition by cabozantinib in prostate cancer PC-3 cells and that this process is regulated by YAP/TBX5 [178].

Studies in patients with primary breast cancer have shown that there is a correlation between the presence of FGFR4 polymorphism (FGFR4-Gly388Arg) and tumor aggressiveness and poor response to adjuvant CMF chemotherapy (cyclophosphamide/methotrexate/5-fluorouracil) but not to endocrine therapy or NCT (neoadjuvant chemotherapy consisting of doxorubicin, cyclophosphamide, docetaxel, and pemetrexed) [179,180,181]. In patients with resected colon cancer (CC) or gastric cancer (GC), the R388 allele was associated with worse overall survival than the G388 allele, especially when fluorouracil/leucovorin or no adjuvant chemotherapy (in CC) or oxaliplatin (in GC) was used [182,183]. In contrast, HNC (head and neck cancer) and ovarian cancer patients carrying the FGFR4-R388 mutation exhibited increased sensitivity to cisplatin treatment, prolonged progression-free and overall survival [184,185]. This diversity is not fully understood, but the R388 allele correlates with increased cell motility and metastasis in many cancers [181,182,183,186,187]. In CC patients the presence of the R388 allele correlated with changes in EMT markers (increased vimentin and Twist, decreased E-cadherin), with concomitant upregulation of AKT, ERKs, and STAT3, compared with patients carrying the G388 allele (Figure 3) [182]. Similarly in NSCLC, FGFR4-R388 led to activation of MAPKs and STAT3, which in turn induced expression of EMT-related genes, including Twist1, N-cadherin, Snail1, and vimentin [187]. STAT3 inhibition in FGFR4-R388 GC cells contributed to decreased vimentin and increased E-cadherin levels, suggesting that the FGFR4-R388/STAT3 pathway plays a key role in EMT of these cells [183]. Interestingly, Whittle and co-workers showed that degradation and internalization of the FGFR4-R388 is slower than that observed for FGFR4-G388 [188]. One mechanism may be the formation of a complex of FGFR4-R388 variant with membrane type 1 matrix metalloproteinase (MT1-MMP) (Figure 3), which controls many cellular functions through proteolytic and non-proteolytic interactions with membrane-associated proteins [189]. MT1-MMP has been identified as a mediator of chemoresistance and modulator of DNA damage response in breast cancer [190]. The MT1-MMP/FGFR4-R388 interaction was found to increase MT1-MMP phosphorylation, endosomal stabilization, and decrease lysosomal degradation of MT1-MMP, which in turn enhances FGFR4-R388 autophosphorylation [189]. Furthermore, increased FGF1-induced MT1-MMP expression was observed in prostate cancer and was found to be dependent on the STAT3 pathway [191]. In another study, the expression of MT1-MMP expression in pancreatic cancer cells was induced by FGF10, which was also correlated with increased cell migration and invasion [192]. In contrast, in MCF-7 cells, MT1-MMP exhibited the opposite effect and downregulated FGF2-dependent signaling by attenuating FGF2/FGFR binding [193].

## 9. Sensitization of Tumor Cells to Chemotherapy by Inhibition of FGF/FGFR Complex Activity

**Figure 4:**
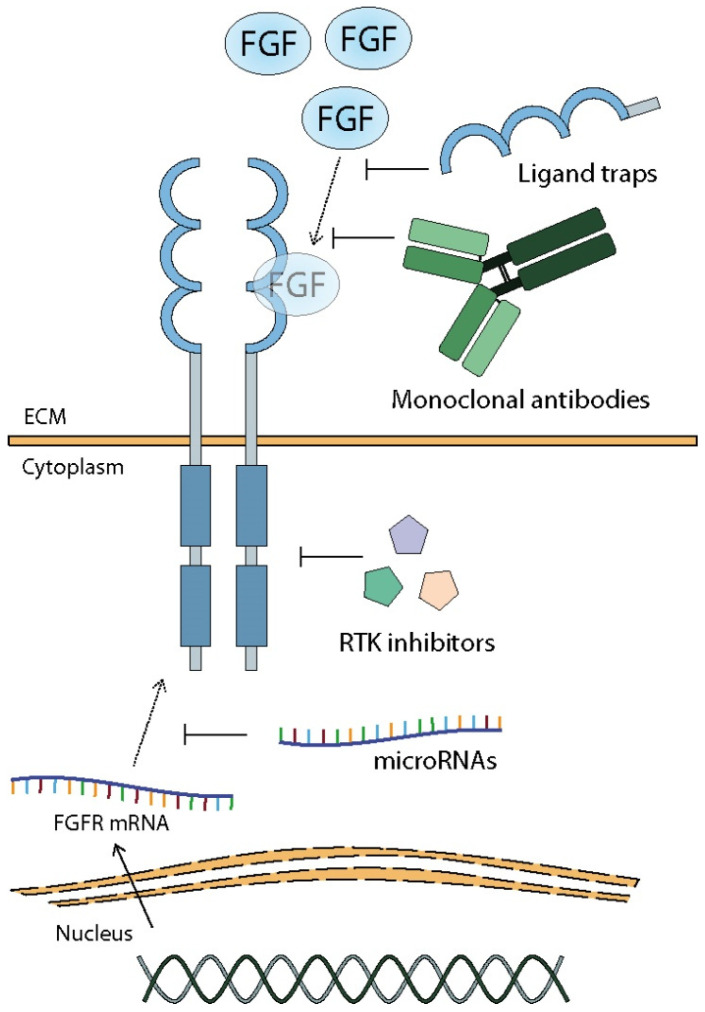
Anti-cancer therapy strategies based on targeting of FGF/FGFR. Ligand traps are fusion molecules that prevent FGF binding and subsequent receptor dimerization. Monoclonal antibodies neutralize specific members of the FGF family members. Chemical receptor tyrosine kinase inhibitors (TKi) are able to inhibit FGFR activation and phosphorylation. Finally, microRNA molecules are capable of silencing both oncogenes and tumor suppressors. Targeting the FGF/FGFR axis with these agents may hold promise for anti-cancer treatment, including overcoming chemoresistance.

Mutations and alterations on the FGF-FGFR axis have been reported in many, including resistant, cancer types. Therefore, a new opportunity to develop personalized therapy based on FGFR-targeting has emerged. In recent years, tremendous progress has been made in modulating or correcting aberrant FGF/FGFR signaling [12,105,194,195]. Such a strategy includes the application of receptor inhibitors, ligand traps, monoclonal antibodies, microRNAs, and combination therapy (Figure 4) [12,105,194,195,196].

### 9.1. TK Inhibitors

Currently, some TKIs have been developed to inhibit FGFR, that can be divided into two groups: (i) non-selective FGFR TKIs, targeting a wide range of tyrosine kinases, including FGFR, and (ii) selective FGFR TKIs, targeting specifically the FGFR group (Table 2) [194,195,196,197]. AZD4547, as an example of the second group, exhibits pro-apoptotic and anti-proliferative activity in cell lines with deregulated FGFR expression, such as KG1a (acute myeloid leukemia line), Sum52-PE (breast cancer cell line), and KMS11 (multiple myeloma line) [198]. Simultaneous treatment with AZD4547 and PLX51107 (BET inhibitor) was shown to counteract FGF2-induced resistance to PLX51107 and to suppress the growth of uveal melanoma tumors [199]. Simultaneous treatment of NSCLC cells with AZD4547 and gefitinib prevented the formation of gefitinib-resistant clones [82]. Similarly, PD173074 was shown to re-sensitize NSCLC to gefitinib treatment [83] and esophageal squamous cell carcinoma (ESCC) to lapatinib treatment [200]. It was also able to prevent proliferation of SCLC lines (H-510 and H-69) in a dose-dependent manner and counteracted FGF2-induced resistance to cisplatin [201]. In HNSCC cells resistant to bevacizumab or pemetrexed-resistant lung adenocarcinoma cells, PD173074 showed potent inhibition of tumor growth and re-sensitization of cancer cells to these drugs [53,176]. Additionally, PD173074 also potentiated the effect of doxorubicin and paclitaxel in endometrial cancer cells carrying a mutation in FGFR2 [202]. Treatment of ER+ breast cancer cells with FGFR inhibitors AZD4547 and PD173074 sensitized the cells to the anti-estrogen tamoxifen suggesting that targeting FGF10/FGFR2 may be a new approach to overcome resistance to hormone-deprivation therapy [203].

Furthermore, PD173074 was able to downregulate P-gp (P-glycoprotein 1) and MRP7 (multidrug resistance protein 7), which in turn increased drug accumulation inside cancer cells and enhanced the toxicity of drugs, such as paclitaxel or vincristine [204,205]. Two other FGFR inhibitors, erdafitinib (JNJ-42756493) and ASP5878, can also re-sensitize cancer cells overexpressing P-gp to anti-cancer drugs [153,206]. However, it is still unclear whether the effect of erdafitinib is solely due to FGFR inhibition, as further analysis revealed an interaction between the inhibitor and P-gp [206,207]. ASP5878 was confirmed to have a clinical potential in the treatment of urothelial cancers expressing mutated FGFR3, including gemcitabine- and doxorubicin (adriamycin)-resistant forms [153]. ASP5878 reduced c-MYC protein level in both parental and gemcitabine-resistant urothelial cancer cell lines, suggesting that c-MYC expression may be regulated by the FGFR signaling [153]. Recent studies on lung cancer revealed that c-MYC is a key downstream effector of FGF/FGFR-dependent signaling in response to oxidative stress, and that FGFR inhibition induced apoptosis through c-MYC downregulation [152]. Interestingly, co-expression of FGFR1 and c-MYC resulted in higher sensitivity to FGFR inhibitors [154]. Another interesting compound is infigratinib (BGJ398), which is a selective pan-FGFR inhibitor and a potent anti-cancer drug candidate [208,209,210]. Inhibition of FGFR by BGJ398 resulted in inactivation of AKT and STAT3 and reduced viability of SKOV3ip1 ovarian carcinoma cells [208]. Treatment with BGJ398 enhanced the cytotoxic effect of paclitaxel/carboplatin cytotoxic activity in ovarian carcinoma cells [208]. A similar effect was observed for alofanib, an allosteric FGFR2 inhibitor [211]. The administration of BGJ398 reduced cell viability and enhanced apoptosis in GIST (gastrointestinal stromal tumor) T-1 cell line resistant to imatinib, but not in parental cells [46]. In GIST cells resistant to doxorubicin, the administration of BGJ398 resulted in a delay in DNA repair [212]. BGJ398 was also found to reduce cell viability, induce apoptosis, and increase the cytotoxicity of 5-fluorouracil or oxaliplatin in CRC cells [52]. Simultaneous treatment of EGFR-positive mesenchymal cancer with BGJ398 and an EGFR inhibitor overcame EGFR inhibitor insensitivity by elimination of EGFR mutant drug-tolerant cells, preventing EMT-associated resistance in both in vitro and in vivo models [213]. Another FGFRs inhibitor, LY2874455, re-sensitizes BRAF-mutant melanoma cells to vemurafenib [47]. A study in drug-resistant gastric cancer cell lines revealed that the specific FGFR2 inhibitor, Ki23057, restored the sensitivity of these cells to irinotecan, paclitaxel, and etoposide, but not to oxaliplatin or gemcitabine [214]. The effect of Ki23057 was accompanied by a decrease in ERCC1 (excision repair-cross complementing gene 1) expression level, and an increase in p53 expression level [214].

### 9.2. Monoclonal Antibodies and Ligand Traps

Two classes of drugs associated with FGF/FGFR inhibition, monoclonal antibodies (mAbs) and FGF-ligand traps, characterized by lower toxicity compared to chemical inhibitors, represent an alternative and promising tool in anti-cancer treatment.

The first group, mAbs, are biologically active molecules that bind to a specific target, such as FGFs, FGFRs or even their isoforms, leading to its inactivation [197,215]. Recently, several mAbs directed towards the FGF/FGFR axis have been developed, including burosumab (KRN23), bemarituzumab (FPA144), BAY1179420, MFGR1877S, GAL-F2, R1MAb1 [12]. Bemarituzumab, an mAb targeting FGFR2b that binds specifically to the IgG III region of the receptor, preventing ligand binding and downstream signaling, has been reported to show anti-tumor activity in breast cancer and gastroesophageal adenocarcinoma [12,105]. Furthermore, the mAb MGFR1877S inhibiting FGFR3 dimerization has been shown to have positive results in the treatment of multiple myeloma and solid tumors [12,105]. Additionally, antibodies against FGFR ligands, including anti-FGF2 mAb 3F12E7, and antibodies targeting FGF8b or FGF19, are being investigated as therapeutic approaches to inhibit tumor growth [12,215].

A second, rapidly developing approach uses ligand traps, fusion molecules that prevent the ligand binding to the receptor. Current FGF-targeting drugs include FP-1039 (GSK3052230), SM27, NSC12, sFGFR2IIIc, sFGFR3, and peptide P3 [12,105]. For example, FP-1039 is an FGF-ligand trap that consists of the extracellular domain of FGFR1 fused to the Fc region of IgG1, capable of binding and neutralizing multiple FGFs such as FGF1, FGF2, and FGF4 [12,197,215,216,217]. This ligand trap has been shown to be able to block FGF2-dependent cell proliferation and inhibit the growth of several cancers in xenograft models, including FGFR1-amplified lung cancer and FGF2-overexpressing mesothelioma [216]. In addition, FP-1039 reduces plasma level of FGF2 in cancer patients in whom standard therapy has been ineffective [216]. Another example is NSC12, which in addition to being a trap for multiple FGFs, can also modulate the formation of FGF/FGFR complex with heparan sulphate proteoglycans, and has been linked to inhibition of lung cancer growth and metastasis [12].

As mAbs and ligand traps are relatively new forms of anti-cancer drugs and their clinical application is limited, there are no reports to date of their action in counteracting drug resistance. However, suramin, a small-molecule compound that acts similarly to ligand traps by preventing growth factors, including FGFs, from binding to receptors [29,218,219], has been shown to significantly reverse FGF-induced resistance and enhance the anti-tumor effect of doxorubicin in the human prostate PC3 cancer model, leading to complete inhibition of tumor growth [218]. It should be mentioned here that another study in NSCLC patients receiving paclitaxel or carboplatin did not confirm the positive effect of suramin (at non-toxic doses) on tumor sensitization to treatment [220].

### 9.3. MicroRNAs

A new approach to anti-cancer therapy and overcoming drug resistance is the administration of microRNAs (miRNAs) [221]. In cancer progression and the development of drug resistance, specific miRNAs, which regulate various cellular processes by silencing the expression of genes, both oncogenes and tumor suppressors, may be down- or upregulated [221,222,223]. For example, miR-205, an FGF2- and VEGFA-targeting molecule that negatively regulates their expression, is highly expressed in breast cancer sensitive to TAC (taxol, doxorubicin, cyclophosphamide) chemotherapy, in contrast to drug-resistance cell lines, in which miR-205 is downregulated [223]. In temozolomide-resistant glioma cells, upregulation of FGFR1 was found to cooperate with downregulation of miR-3116, a molecule silencing the FGFR1 [57]. Re-introduction of miR-3116 resulted in the decrease in FGFR1 level, inhibited the PI3K/AKT pathway, and re-sensitized cancer cells to treatment [57].

All abovementioned strategies strongly demonstrate that targeting the FGF/FGFR axis by different classes of molecules may be a promising strategy for cancer treatment, reducing cancer cell growth, and overcoming chemoresistance (Table 2).

**Table 2:** Anti-cancer strategies overcoming drug-resistance in FGFR-positive tumors.

Class of Drug	Name	Eliminating Resistance to	Cancer Type	References
Chemical inhibitors	AZD4547	Gefitinib	NSCLC	[82]
		PLX51107	Melanoma	[199]
		Tamoxifen	Breast cancer	[203]
	PD173074			
		Gefitinib	NSCLC	[83]
		Lapatinib	ESCC	[200]
		Cisplatin	SCLC	[201]
		Bevacizumab	HNSCC	[53]
		Pemetrexed	Lung cancer	[176]
		Doxorubicin	Endometrial cancer	[202]
		Paclitaxel		
			NSCLC	[205]
			Epidermoid carcinoma	[204]
		Vincristine		
	Erdafitinib (JNJ-42756493)	Colchicine		[206]
	ASP5878	Gemcitabine	Urothelial cancer	[153]
		Doxorubicin		
	BGJ398 (Infigratinib)	Paclitaxel/carboplatin	Ovarian cancer	[208]
		5-fluorouracil	Colorectal cancer	[52]
		Oxaliplatin		
		Imatinib	GIST	[46]
		Doxorubicin		[212]
		Gefitinib	NSCLC	[213]
	Alofanib	Paclitaxel/carboplatin	Ovarian cancer	[211]
	LY2874455	Vemurafenib	Melanoma	[47]
	Ki23057	Irinotecan	Gastric cancer	[214]
		Paclitaxel		
		Etoposide		
Ligand trap	Suramin	Doxorubicin	Prostate cancer	[218]
miRNAs	miR-205	Paclitaxel/doxorubicin/cyclophosphamide	Breast cancer	[223]
	miR-3116	Temozolomide	Glioma	[57]

## 10. Limitations of FGFR Inhibition Therapy—FGFR Mutations and Molecular Cross-Talks with Other Protein

**Figure 5:**
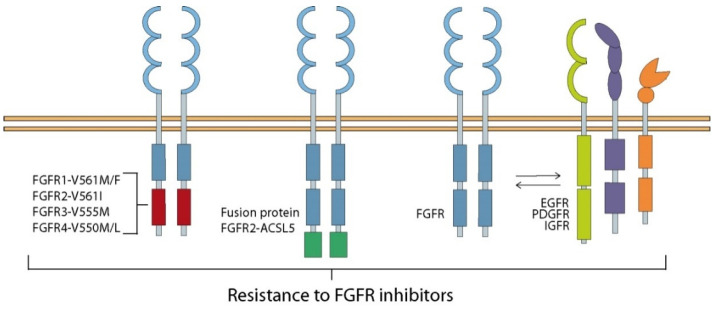
Mutations and molecular cross-talks in the acquisition of resistance to FGFR inhibitors. The use of TKIs in cancer cells expressing FGFRs leads to the acquisition of gatekeeper mutations in the FGFR kinase domain (FGFR1-V561M/F, FGFR2-V561I, FGFR3-V555M, and FGFR4-V550M/L), which in turn desensitize cells to the inhibitor used and may also induce cross-resistance to other inhibitors. FGFR2-ASCL5 fusion leads to the development of resistance to LY2874455, an FGFR inhibitor that overcomes resistance caused by gatekeeper mutations. FGFR inhibition can also lead to the activation of other RTKs, including EGFR, PDGFR, and IGFR, which alternatively trigger downstream cell signaling and render cells insensitive to TKIs.

Despite the proven anti-tumor activity of FGFRs inhibitors, the emergence of new mutations must be taken into account, as well as the existence of molecular cross-talk between the FGFs/FGFRs axis and other proteins, especially other RTK (Figure 5).

Clinical trials have shown that a significant fraction of patients with FGFR-positive tumors do not respond to treatment with FGFRs inhibitors [224,225]. Mutations located in ATP-binding sites of the kinase domain of FGFR have been shown to be a common cause of inhibitor resistance in FGFR-dependent tumors, especially gatekeeper mutation resulting in increased receptor activity [195,196,226,227,228]. Generated gatekeeper FGFR3-V555M mutation in KMS-11 myeloma cells with acquired resistance to AZ12908010 caused cross-resistance to two other FGFR inhibitors, AZD4547 and PD173074 [227]. Byron and co-workers identified 14 mutations in the FGFR2 kinase domain responsible for resistance to dovitinib, which with one exception also provided cross-resistance to PD173074 but not to ponatinib [226]. Only the gatekeeper FGFR2-V565I mutation confers resistance to both inhibitors. A similar mutation was observed for FGFR1 (FGFR1-V561M) in lung cancer, which drives resistance to AZD4547 through the activation of STAT3 and EMT [229]. However, a third generation FGFR inhibitor, GZD824, overcomes resistance caused by FGFR1-V561M/F mutation [230]. Point mutations in the FGFR2 kinase domain were also found in FGFR2 fusion-positive intrahepatic cholangiocarcinoma (ICC) [231,232]. FGFR2 gene fusions with BICC1, AHCYL1, TACC3, MGEA5, and PPHLN1 result in chimeric proteins that can dimerize and activate independently of ligand binding but are still sensitive to FGFR inhibitors.

Recently, clinical trials have also shown a positive effect in ICC patients after treatment with BGJ398 [231]. Unfortunately, in some patients the acquisition of secondary mutations has led to further tumor progression and drug resistance [231,233]. However, additional studies in ICC patients resistant to BGJ398 revealed that an irreversible FGFR inhibitor, TAS-120, eradicated drug resistance [233]. Additionally, in a myeloma cell line resistant to AZ12908010 with a gatekeeper mutation in the FGFR3 gene, a secondary gatekeeper mutation was identified, resulting in the development of cross-resistance to two other FGFR inhibitors, AZD4547 and PD173074 [227]. Gatekeeper mutations in the kinase domain (V550) and hinge-1 (C552) of FGFR4 are acquired as a mechanism of resistance to fisogantib, a selective FGFR4 inhibitor, in HCC [41]. Interestingly, a gatekeeper-agnostic, LY2874455, decreased HCC xenograft growth in the presence of these mutations, demonstrating continued FGF19–FGFR4 pathway dependence [41]. Moreover, FGFR2-ACSL5 fusion, newly identified in GC, led to acquired resistance to LY2874455, highlighting the need to study FGFR2 amplification in terms of developing drug resistance [234].

Another noteworthy aspect is the interaction between FGF/FGFR and other proteins, which may serve cancer cells as an alternative to activate mutagenic cell signaling pathways, including by passing the action of FGFR inhibitors. FGFRs have been identified as co-partners for many cell membrane-associated proteins, including RTKs and G-protein-coupled receptors (GPCRs) [235]. Adachi and co-workers identified a role for other receptor tyrosine kinases such as EGFR, PDGFRα (platelet-derived growth factor receptor α), and IGFR (insulin-like growth factor receptor) in the emergence of resistance to FGFR inhibitor treatment in FGFR1-amplified lung cancer [236]. Combination therapy of BGJ398 with lapatinib in the HCC95 cell line or with linsitinib (an IGFR inhibitor) in the DMS114 cell line was necessary for the downregulation of FRS2α to suppress reactivation of both AKT and ERK and the subsequent induction of apoptotic proteins BIM and PUMA (p53 upregulated modulator of apoptosis) [236]. Interestingly, the acquisition of resistance to BGJ398 in DMS114 cells and urothelial carcinoma cells (RT112) was mediated by the AKT pathway [237]. Monotherapy of BGJ398 or imatinib, a PDGFR inhibitor, resulted in phosphorylation of the second receptor and activation of the downstream MAPK pathway, and only administration of both drugs induced complete inactivation of ERK, indicating an interaction between these receptors, which was confirmed by pull-down [236]. Similarly, in FGFR2-positive GC, resistance to AZD4547 was abolished by EGFR, HER3, or MET inhibition, indicating that these other RTKs are responsible for cancer cell resistance to FGFR inhibition [238]. Acquisition of resistance to infigratinib, a promising FGFR inhibitor used to treat HCC, was associated with elevated HER2 and HER3 levels along with increased enhancer of zeste homolog 2 (EZH2) expression [239]. In HER2-positive breast cancer cells with EMT, resistance to HER2 inhibitor was mediated by increased expression and direct interaction of FGFR1 and neuropilin-1 (NRP1) [175]. Silencing of NRP1 reduced FGF2-dependent ERK activation and inhibited cancer cell growth [175]. Interestingly, NRP1 expression and subsequent drug resistance was mediated by two EMT-related transcription factors, Twist1 and BRD4 [175]. A study by Wang and colleagues linked the activation of HER2/3 to the development of resistance to FGFR inhibitors such as BGJ398 and ponatinib in cell lines harboring FGFR3 amplification [240]. Moreover, FGFR3 was also found as one of the fusion proteins that cause acquired resistance to EGFR inhibitors in lung cancer patients [241].

Another study has shown that the expression of FGF2 by human melanoma cells mediated the promotion of tumor-associated B (TAB) cells to express IGF1 [34]. Co-culture of melanoma cells with TAB cells, but not normal B cells, led to the development of resistance to BRAF and MEK inhibitors as well as cisplatin and paclitaxel treatment. The expression of IGF1 mediated upregulation of FGFR3 and STAT3 in both TAB and melanoma cells. The silencing of IGF1 re-sensitized melanoma cells to BRAF/MEK inhibition, suggesting that IGF1 plays a major role in developing drug resistance in that case. Interestingly, the deactivation of FGFR3 was also able to overcome the resistance indicting the correlation between IGF1 and FGFR3 in the chemoresistance in human melanoma cells. Another notable mechanism of drug resistance related to the FGFR family is that overexpression of FGFRL1 (fibroblast growth factor receptor-like 1) was found in patients with multi-drug resistance SCLC [242]. Although FGFRL1 lacks the kinase domain, there is some evidence indicating the role of FGFRL1 in the regulation of FGF signaling pathways and cancer progression [242,243,244]. In SCLC cells, FGFRL1 was found to interact with ENO1 (alpha-enolase), a protein involved in activation of the PI3K/AKT pathway in tumors, therefore FGFRL1 might modulate drug resistance in SCLC cells [242]. Additionally, it was demonstrated that knockdown of androgen receptor mediated prostate tumor-inducing response of paracrine FGF10, suggesting the role of tumor environment in the initiation of carcinomas and underlying its importance as well as its implications in the development of treatment strategies [245].

## 11. Enhancement of Chemotherapy by FGFs/FGFRs Action

The protective properties of FGFs and their receptors have been widely described, nevertheless, several lines of evidence point to their opposing effects. Ohashi and co-workers found a correlation of FGFR2 lower expression with increased cancer cell proliferation and poor prognosis in patients with gliomas [19]. In MCF-7 breast cancer cells, FGF2 not only inhibited cell growth and proliferation but also potentiated the effects of anti-cancer drugs by downregulation of BCL2 and upregulation of BAX level, thereby enhancing apoptosis [246,247]. Coleman and co-workers suggested that FGF2-induced sensitization to anti-cancer treatment is independent of increased proliferation, S-phase of cell cycle, or p53 activity [248]. FGF2, but not FGF1, was shown to enhance cisplatin toxicity in MCF-7 breast cancer cells and A2780 ovarian cancer cells, but not in SKOV3 ovarian cancer cells or a panel of pancreatic cancer cell lines. In this work, FGF2 did not sensitize cells to etoposide, however, in the other study Wang and co-worker observed reduced colony formation and increased apoptosis in MCF-7 cells induced by FGF2 upon treatment with etoposide or 5-fluorouracil [247,248]. Moreover, in the mouse myoblast cell line C2C12, FGF2 through NFκB activity, but not the PI3K pathway, increased the expression of uridine phosphorylase 1, the enzyme that activates 5-fluorouracil, which was correlated with enhanced toxicity of the drug [249].

In another study, the interaction of FGFR4 with the βKlotho (KLB) co-receptor, a metabolic regulator that is frequently disrupted in hepatic cancers, was shown to inhibit cell proliferation and induce caspase-3-dependent apoptosis in hepatomas by decreasing AKT and mTOR activity [250]. Similarly, KLB expression was downregulated in prostate cancer cells, whereas its overexpression was able to induce apoptosis, inhibit cell proliferation and reverse androgen receptor-dependent and -independent EMT [251]. Furthermore, in breast cancer patients receiving radiochemotherapy (5-fluorouracil, vinorelbine, radiotherapy), FGFR1 expression correlated with good response to treatment, whereas FGFR1-negative cancers showed resistance to this treatment [252]. Additionally, the reduced FGF14 level was correlated with poor survival and oncogenic mutation status (e.g., KRAS, EGFR) in lung adenocarcinoma (LUAC) patients [253]. Overexpression of FGF14 in NSCLC LUAC cell line (A549) resulted in decreased proliferation, colony formation and migration, and enhanced mesenchymal to epithelial transition, indicating that FGF14 reduces the invasiveness of lung cancer cells in vitro while ablation of FGF14 in these cells reversed the above changes, supporting its suppressive role in lung cancer progression. Furthermore, RNA sequencing data suggested that genes affected by FGF14 were associated with the extracellular matrix (upregulation of *Ccbe1* and *Adarb1*, downregulation of *Coll11a1* and *Muc16*), and thus could play a role in proliferation and migration. Therefore, therapeutics activating the tumor suppressive properties of FGF14 would be a promising strategy for the treatment of LUAC patients.

## 12. Concluding Remarks

Due to multiple critical biological activities, the FGF/FGFR axis plays an important role in cancer growth and progression. Chromosomal abnormalities often lead to overexpression of FGFs and their receptors, formation of fusion proteins, and generation of constitutively active kinase domains that can deregulate downstream signaling and further promote uncontrolled proliferation, apoptosis avoidance, and metastasis. However, the role of FGF/FGFR in carcinogenesis extends beyond cancer development and progression, as increasing evidence suggests a close link between FGF/FGFR signaling and the failures of various currently available anti-cancer therapies. To date, several distinct mechanisms of FGF/FGFR-mediated anti-cancer drug resistance have been described, but the picture is far from complete, highlighting the need for further studies in this field.

Although many drugs (such as paclitaxel, cisplatin, etoposide, or doxorubicin) show promising anti-cancer activity, the acquisition of drug resistance complicates the treatment. The reduced responses to therapeutic agents have been associated with the deregulation of several signaling pathways, such as PI3K/AKT, MAPKs, STATs, and PLCγ/PKC, which can be activated by FGF/FGFR. Not only mutations in signaling proteins and aberrant signal transduction, but also cross-talks with other pathways are major obstacles to overcome acquired resistance. Abnormalities in signal transduction can lead to deregulation of biological process such as apoptosis (through an imbalance between its inhibitors and activators), angiogenesis (through an increase in VEGF expression), and EMT (through disproportion in expression between epithelial and mesenchymal markers), making the development of effective therapy even more challenging [105].

In addition to canonical FGFs, other members of the FGF family may be involved in the acquisition of drug resistance by cancer cells. For example, FGF13, a representative of intracrine FGFs, has been found to contribute to the insensitivity of cervical cancer cells to cisplatin [38]. Recent studies have shown that also FGF12 has anti-apoptotic properties in the osteosarcoma U2OS cell line overexpressing FGFR1 (U2OS-R1), but this has not yet been linked to drug resistance [254].

The interference of multiple factors can result in an impaired cellular response to anti-cancer treatment. For instance, tumor heterogeneity may lead to the development of different drug sensitivities in individual cells as well as the evolution of more resistant clones. Another aspect is the diverse microenvironment of cancer cells, where the presence of various cytokines, growth factors, and extracellular matrix compounds, often derived from CAFs, may cause additional changes in cellular metabolism.

Deregulated metabolism can drive further mutations (e.g., gatekeeper mutations in kinase domain of FGFR) leading to cross-resistance and thus to the ineffectiveness of even the most specialized strategies [195,196,226,227,228]. On the other hand, reports of positive effects of FGF/FGFR on chemotherapy further emphasize the complexity of the whole system [246,247,248].

Finally, interactions of FGF receptors with other proteins, including RTKs, and cross-activation between proteins from independent signaling pathways might prevent effective therapy. A good example is the interaction of FGF/FGFR complex with galectins, which constitute a family of carbohydrate binding proteins that modulate many critical cellular processes [255]. In recent years, galectins have emerged as major determinants of tumor sensitivity or resistance to various anti-cancer therapies [256]. Importantly, the involvement of galectins in the FGF/FGFR signaling was suggested several years ago [257]. We have recently shown that extracellular galectin-1 activates FGFR1 and thereby stimulates cell proliferation and apoptosis avoidance [258]. In addition, galectins may facilitate cross-talk of FGFRs with themselves [259]. The role of galectins in FGFR-dependent chemoresistance has not been demonstrated yet. Since FGFRs and galectins are involved in diverse mechanisms of chemoresistance, it is tempting to speculate that these two groups of proteins, may form a complex network modulating tumor sensitivity to various anti-cancer drugs.

Targeted therapy based on modulation of FGF/FGFR activity using ligand traps, monoclonal antibodies, RTK inhibitors, and microRNAs offers promising, effective therapeutic approaches. Several studies on FGF/FGFR inhibition have demonstrated its efficacy in re-sensitizing cells to treatment. Another interesting therapeutic option to overcome resistance to TK inhibitors is the disruption of lysosome architecture to release sequestrated TK inhibitors [196]. Similarly, the concomitant use of inhibitors to abolish drug resistance has been shown to be more effective in clinical trials [194,260]. Another promising strategy is combination therapy with TKIs and signaling pathway inhibitors [194,237,261,262], as well as simultaneous inhibition of FGFRs and induction of apoptosis [263,264,265]. Ligand traps and mAbs targeting FGFR/FGF are relatively new approaches and are still relatively few in clinical trials. However, they appear tohave great potential to overcome drug resistance, particularly when combined with other therapies, including inhibitor treatment, radiotherapy, or chemotherapy.

In recent years, antibody–drug conjugates (ADCs) have emerged as a precise and powerful tool in cancer treatment [266]. These bioconjugates consist of an mAb that specifically binds a tumor surface antigen and a potent drug, such as monomethylauristatin E, calicheamicin, maytansinoid, or camptothecin [267]. To date, 12 ADCs have been approved by Food and Drug Administration as therapeutics for oncology indications, and many more are currently under investigation [267]. Nevertheless, they usually contain a single cytotoxic drug, which can easily lead to the development of chemoresistance. Therefore, dual-drug conjugates, with two distinct mechanisms of action, showing enhanced efficacy and hindering the development of drug resistance, appear to be the future of anti-cancer strategies [268,269].

Moreover, endocytosis and RTK signaling are closely connected, also in cancer cells [270]. It is plausible that endocytosis, including FGF/FGFR endocytosis, may influence resistance to anti-cancer treatment by modulating the level of FGF/FGFR and/or the specificity and duration of their signaling, and consequently the efficacy of targeted therapies [271,272,273]. An effective solution could be the generation of targeting molecules that, by exploiting multiple endocytosis routes simultaneously, enable continuous delivery of drugs when cancer cells begin to manipulate endocytosis pathways as a defense mechanism [274].

The multitude of alternative strategies being developed in parallel, and the rapid progress of targeted therapies, bring hope in the field of personalized medicine in overcoming drug resistance in various cancers, including FGFR-dependent ones.

## Figures and Tables

**Table 1:** Examples of resistant cancer types associated with FGF/FGFR.

Cancer Type	Drug	Involved Protein(s)	References
Breast cancer	Etoposide	FGF2	[30]
	5-fluorouracil		
	Mifepristone, Telepristone		[50]
	Paclitaxel		[31,33]
	Tamoxifen	FGF1, FGFR2	[48,51]
	Trastuzumab	FGFR4	[49]
		FGF4	[43]
	Lapatinib		
Colorectal cancer	5-fluorouracil	FGFR4	[52]
	Irinotecan	FGF2, FGF9	[37]
Liver cancer	Sorafenib	FGF19, FGFR4	[39]
		FGF9	[42]
Head and neck cancer	Paclitaxel	FGF2	[31]
	Cisplatin	FGF2, FGFR2	[35]
	Bevacizumab	FGF2, FGFR3	[53]
Lung cancer	Gefitinib	FGFR1	[54]
	Cisplatin	FGF2	[36]
	Erlotinib	FGFR1	[45]
Bladder cancer	Cisplatin	FGF2	[27]
	Paclitaxel		[31]
Prostate cancer	Paclitaxel	FGF2	[31]
		FGF1, FGF2	[29]
	Doxorubicin		
	5-fluorouracil		
Blood cancer	Cytarabine	FGF2, FGFR1	[55]
	Etoposide		
	Fludarabine	FGF2	[26]
Ovarian and cervical cancer	Paclitaxel	FGF2	[31]
	Etoposide	FGF1	[56]
	Cisplatin		[32]
		FGF13	[38]
Brain cancer	Temozolomide	FGFR1	[57]
		FGF2	[58]
Melanoma	Paclitaxel	FGF2	[34]
	Cisplatin		
	Vemurafenib	FGFR3	[47]
Bone cancer	Doxorubicin	FGF2	[28]

## Abbreviations

ADC	antibody–drug conjugate
BAD	BCL-2 antagonist of cell death
BAX	apoptosis regulator BAX
BCL-2	B-cell CLL/lymphoma 2
BCL-XL	B-cell lymphoma-extra large
BIM	BCL-2-like protein 11
BRAF	serine/threonine-protein kinase B-raf
CAFs	cancer-associated fibroblasts
CAM	cell adhesion molecule
CC	colon cancer
CK2	casein kinase 2
CMF	cyclophosphamide/methotrexate/5-fluorouracil chemotherapy
CRC	colorectal cancer
DAG	diacyloglycerol
E12/E47	immunoglobulin enhancer-binding factor lub transcription factor E2-alpha
ECM	extracellular matrix
EF1/ZEB1	elongation factor 1/zinc finger E-box-binding homeobox 1
EGF	epithelial growth factor
EGFR	epithelial growth factor receptor
EMT	epithelial-to-mesenchymal transition
ENO1	alpha-enolase
ER	estrogen receptor
ERCC1	excision repair-cross complementing gene 1
ERK	extracellular signal-regulated kinase
ESCC	esophageal squamous cell carcinoma
FGF	fibroblast growth factor
FGFR	fibroblast growth factor receptor
FGFRL1	fibroblast growth factor receptor-like 1
ESCC	esophageal squamous cell carcinoma
FLIP	FLICE-like inhibitory protein
FOXO1	forkhead box protein O1
FRA1	Fos-related antigen 1
FRS2α	fibroblast growth factor receptor substrate 2
GAB1	GRB2-associated binding protein 1
GC	gastric cancer
GIST	gastrointestinal stromal tumor
GPCR	G-protein-coupled receptor
GRB2	growth factor receptor-bound 2
GSK3β	glycogen synthase kinase 3β
HA	hyaluronan
HER2/3	receptor tyrosine-protein kinase erbB-2/3
HES1	hairy and enhancer of split1
HCC	hepatocellular carcinoma
HGF	hepatocyte growth factor
HNC	head and neck cancer
HNSCC	head and neck squamous cell carcinoma
HO-1	hemeoxygenase 1
HSC	hepatic stellate cells
HSP90	heat shock protein 90
IP3	inositol-1,4,5-triphosphate
ICC	intrahepatic cholangiocarcinoma
IGF	insulin-like growth factor
IGFR	insulin-like growth factor receptor
IKK-β	inhibitors of NFκB kinase-β
JAK	Janus kinases
JNK	c-Jun N-terminal kinase
KLB	βKlotho
LUAC	lung adenocarcinoma
mAb	monoclonal antibody
MACOM	MDS1 and EVI1 complex locus protein EVI1
MAPK	mitogen-activated protein kinases
MCL1	myeloid cell leukemia sequence 1
MDM2	mouse double minute 2 homolog
MEK	mitogen-activated protein kinase kinase
MMTV	mouse mammary tumor virus
miRNA	microRNA
MRP7	multidrug resistance protein 7
MT1-MMP	membrane type 1 matrix metalloproteinase
mTOR	mammalian target of rapamycin
mTORC1/2	mammalian target of rapamycin complex 1/2
NCT	neoadjuvant chemotherapy
NFκB	nuclear factor-κB
Notch	neurogenic locus notch homolog protein
NRP1	neuropilin-1
Nrf2	nuclear factor E2-related factor 2
NSCLC	non-small-cell lung cancer
P53	cellular tumor antigen p53
PARP	poly (ADP-ribose) polymerase
PD-ECGF	platelet-derived endothelial cell growth factor
PDGFRα	platelet-derived growth factor receptor α
P-gp	P-glycoprotein 1
PI3K	phosphoinositide 3-kinase
PIP2	phosphatidylinositol-4,5-bisphosphate
PKB	protein kinase B
PKC	protein kinase C
PLA2G4C	phospholipase A2γ
PLCγ	phospholipase Cγ
PPHLN1	periphilin-1
PRKACB	cAMP-dependent protein kinase catalytic subunit beta
PUMA	p53 upregulated modulator of apoptosis
RAP1	RAS-proximate-1 or Ras-related protein 1
RAS	rat sarcoma virus protein
ROS	reactive oxygen species
RTK	receptor tyrosine kinase
S6K2	ribosomal p70 S6 kinase 2 (jest teżskrót S6K1)
SCLC	small cell lung cancer
SIP1/ZEB2	smad interacting protein 1/zinc finger E-box-binding homeobox 2
SNT-1	suc1-associated neurotrophic factor-induced tyrosine-phosphorylated target
SOS1	son of sevenless 1
SOX2	Sry-related HMG box 2
STAT	signal transducers and activators of transcription
TAB	tumor-associated B cells
TAC	taxol, doxorubicin, cyclophosphamide chemotherapy
TGFβ	transforming growth factor β
Tin-PP	Tin-Protoporphyrin
TKI	tyrosine kinase inhibitor
TSC1/2	tuberous sclerosis complex
TYK2	tyrosine kinase 2
UPP1	uridinephosphorylase 1
VEGF	vascular endothelial growth factor
VEGFR	vascular endothelial growth factor receptor
